# A Comparison of ACQ, AIE and AEE-Based Polymers Loaded on Polyurethane Foams as Sensors for Explosives Detection

**DOI:** 10.3390/s18051565

**Published:** 2018-05-15

**Authors:** Zhiwei Chu, Zhuxin Fan, Xiang Zhang, Xiaofeng Tan, Dongxu Li, Guohua Chen, Qinghua Zhao

**Affiliations:** 1 College of Materials Science and Engineering, Huaqiao University, Xiamen 361021, China; chuzhiwei1991@126.com (Z.C.); 1300507012@hqu.edu.cn (Z.F.); 1611302036@hqu.edu.cn (X.Z.); lidongxu@hqu.edu.cn (D.L.); hdcgh@hqu.edu.cn (G.C.); 2 Department of Polymer Chemistry and Technology, Kaunas University of Technology, K. Barsauskog. 59, 51423 Kaunas, Lithuania; xiaofeng.tan.chn@gmail.com

**Keywords:** tetraphenylethane, ultrasonication, quenching, detect explosive, recycle

## Abstract

An aggregation-caused quenching (ACQ)-active polymer (PF), an aggregation-induced emission (AIE)-active polymer (PFTPE) and an aggregation-enhanced emission (AEE)-active polymer (PTTPE) were synthesized by tetraphenylethane (TPE), fluorene and thiophene moieties. Polyurethane (PU) foams modified by PF, PFTPE and PTTPE, namely PU-PF, PU-PFTPE and PU-PTTPE, using ultrasonication-assisted method have been prepared. A comparative study of PU-PF, PU-PFTPE and PU-PTTPE for detection explosives had been performed, and significant fluorescence quenching was observed with the introduction of PA solutions. The as-prepared PU-PF, PU-PFTPE and PU-PTTPE sensors exhibited a superior sensitivity for PA solutions with different concentrations. Remarkably, PU-PF gave a quenching efficiency of 96.2%, higher than 93.5% for PU-PFTPE and 86.7% for PU-PTTPE at a PA concentration of 180 µg·mL^−1^ in methanol, which was attributed to the effective energy transfer from the fluorophore (PF) to the nitro explosive (PA). This suggested that some ACQ polymers, applied to detect explosives, could afford better performances than AIE or AEE polymers through modification of structures and selection of adequate carriers. At the same time, these chemical sensors can be recycled many times.

## 1. Introduction

Significant efforts have been employed to the exploration for detecting explosives [1,2,3,4,5]. Positively, the adverse effects of explosives on national defense security, ecological environment and human health have aroused widespread concern, such as PA (2,4,6-Trinitrophenol), TNT (2,4,6-Trinitrotoluene) and DNT (2,4-Dinitrotoluene). Quintessentially, fluorescence-based chemical sensors with conjugated polymers have appeared as new reliable approaches in recent years for sensing explosives because of their low cost, portability, high sensitivity, rapid response time; as well as, dual applicability in vapor, solid and solution media [6,7,8]. Photoinduced excited electronics transfer (PET) or fluorescence resonance energy transfer (FRET) from the electronic-rich polymers (donor) to electronic-defect nitroaromatic compounds (acceptor), and then fluorescence quenching systems are constructed [9,10,11]. Recently, polyfluorene (PF) and its derivatives have been used as chemosensors, owing to their high photoluminescence (PL) efficiencies [12,13,14]. However, PFs suffer from aggregating and forming excimers in solid states, which result in reducing efficiencies and fluorescence quenching, namely aggregation-caused quenching (ACQ) [4]. Thus, the ACQ-property of polymers urgently need to be changed.

Many approaches have been utilized to hamper these luminophore aggregations in order to inhibit the ACQ effect, through chemical, physical or engineering approaches and processes [15,16]. For example, branched chains or dendritic wedges have been covalently introduced to the structure to obstruct the formation of aggregates [16]. Among the approaches, the introduction of tetraphenylethene (TPE) into polymers is considered to be one of the most valid approaches. TPE is one of the most popular AIE molecules, which are characterized by aggregation-induced emission (AIE) resulted from the propeller-shaped structure. Since it can be easily prepared, large amounts of AIE or AEE materials, based on TPE, have been developed [17,18,19,20,21,22,23,24,25,26,27,28]. Typically, numerous TPE-based polymers, spread in good-poor pair solvents or spun coating films as probes for explosive analysis, have been widely studied [26,27,28]. For example, Li et al. [20] have synthesized a series of TPE-containing conjugated polymers with fluorene or carbazole moieties, and used them to probe an explosive with high sensitivity both in the nanoparticle and solid state. Consequently, some researchers believe that fluorescent polymers with ACQ property for detecting explosives have to suffer from the ACQ effect to cripple the detection results [29,30]. Meanwhile, it seems that AIE or AEE materials could afford better performance to detect explosives than ACQ materials. Unfortunately, there have been few reports focused on the comparison of ACQ, AIE and AEE polymers as probes to detect explosives under the same conditions. Maybe the AIE or AEE materials are better than the ACQ materials?

**Figure 1:**
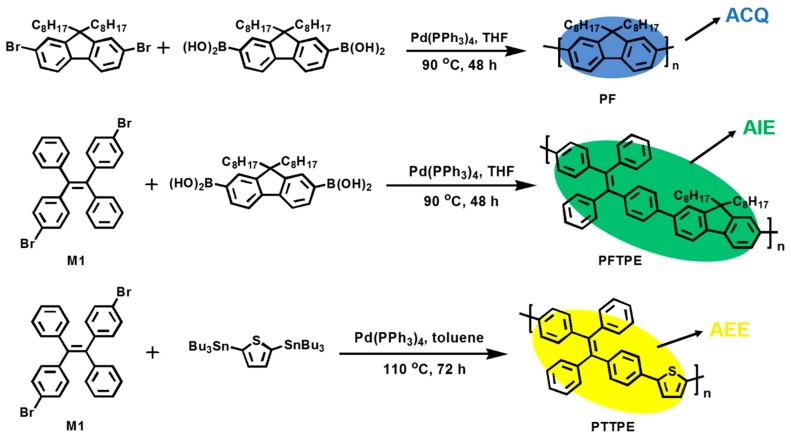
Synthetic routes of PF, PFTPE and PTTPE.

To the best of our knowledge, using nanoaggregates in solvents as probes was not enough to meet practical applications, while it would be more convenient to use the nanoaggregates or solid state loaded on suitable carriers. Polyurethane (PU) sponges were widely used in absorbing water, oils and organic solvents due to the 3D porous structure of PU and the facile attempts reported by literature [31,32,33,34]. In this work, we have synthesized an ACQ polymer (PF), an AIE polymer (PFTPE) and an AEE polymer (PTTPE) with fluorene, TPE, and thiophene moieties by Suzuki coupling reaction. Figure 1 illustrates the synthetic route to PF, PFTPE and PTTPE. Then PU foams modified by these three kinds of polymers, namely PU-PF, PU-PFTPE and PU-PTTPE, were prepared using ultrasonication method. In our previous work, we have reported that an AIE polymer was loaded on PU foams by ultrasonication approach to detect explosives and showed high sensitivity to the PA solution and DNT vapor [35]. The performances of PU-PF, PU-PFTPE and PU-PTTPE utilized to detect explosives have been investigated and compared.

## 2. Materials and Methods

### 2.1. Materials

9,9-Dioctyl-2,7-dibromofluorene, 9,9-Dioctylfluorene-2,7-diboronic acid, 2,5-Bis(tributylstannyl) thiophene and 2-(Tributylstannyl)thiophene were purchased from Sigma-Aldrich, TCI, or Acros Organics. Tetrahydrofuran (THF), bromobenzene and Pd(PPh_3_)_4_ were purchased from SunaTech Inc. 4-Bromobenzophenone, titanium tetrachloride and Zn were purchased from Energy Chemica. The nitro explosive analytes used in experiments were purchased from Aldrich and used as received. Other chemicals were used as received unless otherwise specified.

### 2.2. Measurements and Characterization

The ^1^H NMR and ^13^C NMR spectra were recorded with a Nuclear magnetic resonance spectrometer (Bruker AVANCE III 500 MHz). The thermal analyses were performed on Shimadzu DTG-60H thermogravimetric analyzer, in a nitrogen atmosphere at a rate of 10 °C/min. Differential scanning calorimetry (DSC) was conducted under nitrogen on TA DSC2910/SDT2960, and the sample was heated at a temperature of 20 °C/min from 30 °C to 250 °C. UV-vis absorption spectra and PL spectra were measured by Shimadzu UV-3100 spectrophotometer and Edinburgh FL/FS920 TCSPC luminescence spectrophotometer, respectively. Molecular weights and polydispersities of the copolymers were determined by gel permeation chromatography (GPC) analysis, with polystyrene standard calibration (waters high-pressure GPC assembly Model M515 pump, l-Styragel columns of HR4, HR4E, and HR5E with 500 and 100 Å, refractive index detectors, solvent THF). Cyclic voltammetry (CV) experiments were carried out using a PARSTAT 2273 electrochemical analyzer, with a three-electrode cell in a solution of Bu_4_NBF_4_ (0.1 M) in acetonitrile, at a scan rate of 100 mV/s. A Pt wire was used as the counter electrode, and an Ag/AgCl (0.1 M) electrode was used as the reference electrode. Prior to each series of measurements, the electrolytic cell was deoxygenated with nitrogen. The morphologies of modified PU foams were characterized by field emission scanning electron microscopy (FESEM, Hitachi SU70). Fluorescence microscope photos were taken by Zeiss Axio Imager A1.

### 2.3. Synthesis of PF, PFTPE and PTTPE

#### 2.3.1. Synthesis of Poly(2,7-9,9′-dioctyl-9H-fluorene) (PF)

In a 2-necked, 50 mL flask, a stirred solution of 9,9-dioctyl-2,7-dibromofluorene (0.548 g, 1 mmol) and 9,9-dioctylfluorene-2,7-diboronic acid (0.574 g, 1.2 mmol) dissolved in THF (20 mL), then K_2_CO_3_ (2 M, 10 mL), Pd(PPh_3_)_4_ (0.035 g, 3 mmol%) were added. After being refluxed for 48 h, phenylboronic acid and bromobenzene were added for end capping. 10 h later, the reaction mixture was extracted for three times with CH_2_Cl_2_ and dried over MgSO_4_. Then precipitation was performed twice with dichloromethane/methanol, PF was obtained with a yield of 90%. ^1^H NMR (500 MHz, CDCl_3_, δ): 8.28–7.80 (2H, m), 7.82–7.59 (2H, m), 7.58–7.30 (2H, m), 2.41–1.90 (4H, m), 1.87–1.02 (24H, m), 1.01–0.58 (6H, m) (Supplementary Materials Figure S1). ^13^C NMR (125 MHz, CDCl_3_, δ): 152.46, 139.96, 130.08, 126.07, 121.53, 119.77, 55.39, 40.24, 31.84, 31.51, 29.78, 29.27, 23.69, 22.63, 14.13 (Supplementary Materials Figure S2). M_w_ = 1.22 × 10^4^, M_w_/M_n_ = 2.57.

#### 2.3.2. Synthesis of Poly((E)-1,2-diphenyl-1,2-di-p-tolylethene-alt-9,9′-dioctyl-fluorene) (PFTPE)

Br-TPE-Br was synthesized according to the literature [36]. In a 2-necked, 50 mL flask, a stirred solution of Br-TPE-Br (0.49 g, 1 mmol) and 9,9-dioctylfluorene-2,7-diboronic acid (0.574 g, 1.2 mmol) dissolved in THF (20 mL), then K_2_CO_3_ (2 M, 10 mL), Pd(PPh_3_)_4_ (0.035 g, 3 mmol%) were added. After refluxed for 48 h, phenylboronic acid and bromobenzene were added for end capping. 10 h later, the reaction mixture was extracted for three times with CH_2_Cl_2_ and dried over MgSO_4_. Then precipitation was performed twice with dichloromethane/methanol, PFTPE was obtained. Yield: 88%. ^1^H NMR (500 MHz, CDCl_3_, δ): 7.85–7.75 (2H, m), 7.70–7.59 (4H, m), 7.60–7.46 (3H, m), 7.46–7.25 (6H, m), 7.25–7.13 (7H, m), 7.14–6.85 (2H, m), 2.23–1.80 (4H, m), 1.90–1.03 (24H, m), 1.02–0.56 (6H, m) (Supplementary Materials Figure S3). ^13^C NMR (125 MHz, CDCl_3_, δ): 156.08, 153.26, 151.72, 144.05, 143.44, 142.88, 141.29, 140.73, 140.28, 139.48, 133.15, 131.45, 129.45, 129.02, 128.60, 128.05, 127.51, 126.29, 125.92, 121.08, 120.05, 55.34, 40.57, 31.88, 30.14, 29.86, 29.33, 23.86, 22.71, 14.21 (Supplementary Materials Figure S4). M_w_ = 1.64 × 10^4^, M_w_/M_n_ = 3.80.

#### 2.3.3. Synthesis of Poly((E)-1,2-diphenyl-1,2-di-p-tolylethene-alt-2,5-thiophene) (PTTPE)

Br-TPE-Br was synthesized according to the literature [36]. In a 2-necked 50 mL flask, a stirred solution of Br-TPE-Br (0.49 g, 1 mmol) and 2,5-Bis(tributylstannyl)thiophene (0.794 g, 1.2 mmol) dissolved in toluene (30 mL), then K_2_CO_3_ (2 M, 10 mL), Pd(PPh_3_)_4_ (0.035 g, 3 mmol%) were added. After refluxed for 72 h at 110 °C, bromobenzene and 2-(Tributylstannyl)thiophene were added for end capping. 10 h later, the reaction mixture was extracted for three times with CH_2_Cl_2_ and dried over MgSO_4_. Then precipitation was performed twice with dichloromethane/methanol, PFTPE was obtained. Yield: 63%. ^1^H NMR (500 MHz, CDCl_3_, δ): 7.48–7.29 (4H, m), 7.27–7.19 (2H, m), 7.19–7.10 (8H, m), 7.10–6.96 (6H, m) (Supplementary Materials Figure S5). ^13^C NMR (125 MHz, CDCl_3_) δ (ppm): 143.64, 143.54, 143.21, 143.00, 142.92, 140.62, 132.39, 132.24, 131.94, 131.46, 127.95, 127.75, 126.74, 126.61, 124.82, 124.64, 123.88 (Supplementary Materials Figure S6). M_w_ = 9.99 × 10^3^, M_w_/M_n_ = 2.79.

## 3. Results and Discussion

### 3.1. Optical Properties of PF, PFTPE and PTTPE

**Figure 2:**
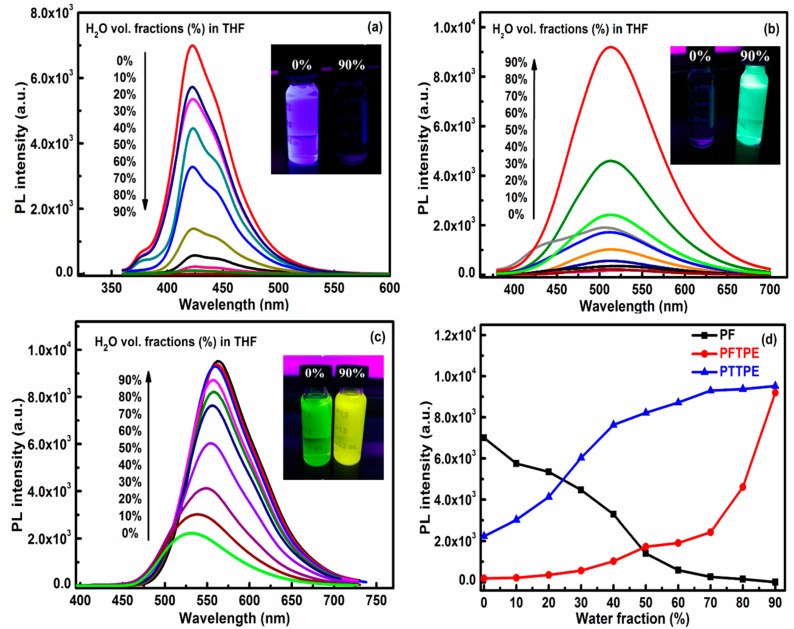
PL spectra of PF (**a**), PFTPE (**b**) and PTTPE (**c**) (100 µg·mL^−^^1^) in H_2_O/THF mixtures with different water fractions (*f*_w_), inserts: photographs of PF (**a**), PFTPE (**b**) and PTTPE (**c**) in H_2_O/THF (*f*_w_ = 0 and 90 vol%), taken under the illumination of the UV lamp (λ = 365 nm); (**d**) PL spectra of PF, PFTPE and PTTPE (100 µg·mL^−^^1^) in H_2_O/THF mixtures with different water fractions (*f*_w_).

As mentioned above, materials with the performance of AIE or AEE could be obtained by introducing the TPE unit to fluorene and thiophene moieties. Thus, we investigated their PL behaviors of PF, PFTPE and PTTPE in solution and aggregate states. During the preparation of the different water concentrations of mixed solution, firstly, respectively divided the three polymer solutions (1000 µg in 10 mL THF) into ten equal parts in 10 mL bottles, then we added the distilled water and THF into their THF solutions to 10 mL under shaking evenly. The resultant mixtures were visually uniform and transparent, indicating that PF, PFTPE and PTTPE aggregates were nanometer-sized [21,37]. PF showed a strong fluorescence at 425 nm in pure THF as well as the fluorescence intensity gradually weakened with increasing water content; whereas, PF almost did express none fluorescence while containing no THF under the UV lamp (λ = 365 nm) (Figure 2a). Thus, these phenomena confirmed that PF possessed ACQ characteristic.

On the other hand, as shown in Figure 2b, PFTPE depicted a maximum emission peak at 515 nm, which was red-shifted than PF due to the more conjugated effect. In contrast, PFTPE was almost non-emissive while dissolving in pure THF under the UV lamp (λ = 365 nm). With the gradual addition of the distilled water into THF, the PL intensities of PFTPE were progressively enhanced and the PL peak invariably centered at 515 nm, indicating that the polymer exhibited a remarkable AIE feature due to the TPE residue. Obviously, aggregation of its chains should occur readily in THF/H_2_O mixtures with high water contents since it was insoluble in water.

Different from PF and PFTPE, PTTPE existed an obvious fluorescence emission peak at 525 nm in pure THF and transferred peaks to 563 nm with the distilled water gradually added to THF. During this process, the fluorescence intensity enhanced gradually. The specific changes were indicated in Figure 2c,d, which stating clearly that PTTPE exhibited a remarkable AEE property due to the TPE residue. It was AEE active because the emission of the polymer in pure THF was not completely quenched. The emission pictures of PTTPE under different water contents was shown in the Supplementary Materials Figure S7. Compared with the previous two polymers, the PTTPE had a red-shift evidently, which due to the increase of the conjugation length and the properties of thiophene.

### 3.2. Thermal Properties of PF, PFTPE and PTTPE

**Figure 3:**
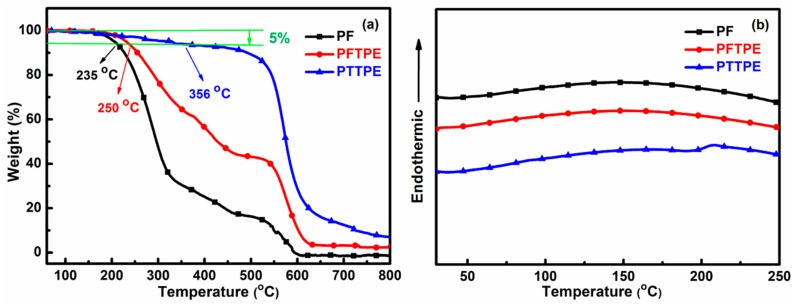
TGA (**a**) and DSC (**b**) curves of PF, PFTPE and PTTPE.

Thermal stability of the polymers was an important parameter with regard to real-world applications. Thus, the thermal stabilities of PF, PFTPE and PTTPE were recorded by thermogravimetric analysis (TGA) and differential scanning calorimeter (DSC) (Figure 3). The decomposition temperatures for a 5% weight loss (T_d_) of these three polymers severally were up to 235 °C, 250 °C and 356 °C. At the same time, the DSC curves of PF, PFTPE and PTTPE did not detect obvious peaks corresponding to glass transitions, which indicated their good thermal stability. Herein, compared these three polymers, PFTPE and PTTPE showed better thermal stability due to the increase of rigid aromatic rings.

### 3.3. Preparation of Functional Sensors

Ultrasonication-assisted manufacture is a common method in scientific research, which has been used in many fields such as crushing, rinsing, emulsification and activation of particles [37,38,39], due to providing a strong interaction between energy and matter. To understand more about the manufacture approach, our groups have tried to prepare PU foams modified by polymers through the ultrasonication method, as we have reported previously [35]. The advantages of ultrasonication-assisted method could be discovered by comparing with the immersing method because the nanoparticles could be firmly adhered onto the PU foam skeleton with high speed and energy under the treatment of ultrasonication [40,41,42]. Therefore, PU-PF, PU-PFTPE and PU-PTTPE were prepared with these three polymers’ nanoparticles at 100 µg·mL^−1^ in mixed solution (volume, THF/H_2_O = 1/9) through the ultrasonication method.

**Figure 4:**
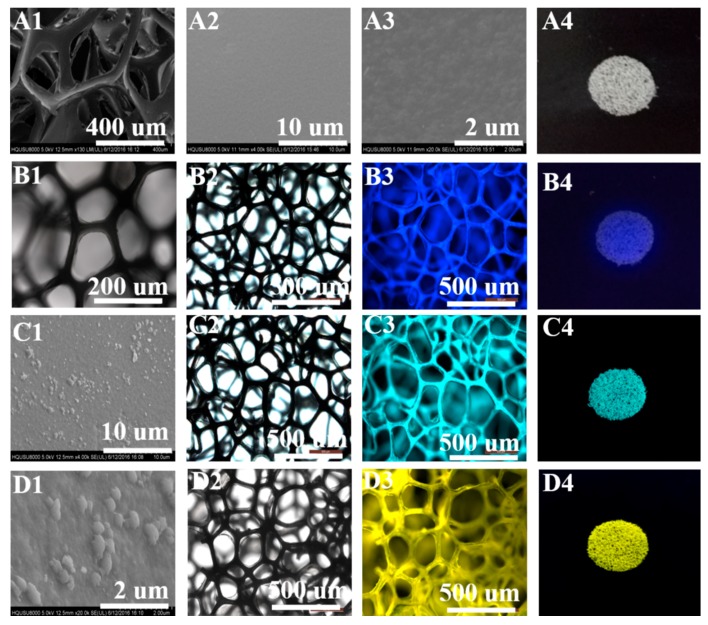
SEM images of pure foams (**A1**–**A3**) and loaded foams (**B1**,**C1**,**D1**); Fluorescence microscope photos of PU-PF (**B2**,**B3**), PU-PFTPE (**C2**,**C3**) and PU-PTTPE (**D2**,**D3**); Photo of pure foam (**A4**); Photos of PU-PF (**B4**), PU-PFTPE (**C4**) and PU-PTTPE (**D4**) under the UV lamp (λ = 365 nm).

In order to evaluate the effect of nanoparticles on the PU foams, we observed the sponge skeleton through FESEM and fluorescence microscope. Remarkably, the pure PU foam possessed a smooth surface, which could be seen from the SEM images in Figure 4(A1–A3). However, in contrast to the photographs in Figure 4(B1,C1,D1), the modified PU foam surface was covered with polymer nanoparticles. In addition, the normal PU foams without anything polymer nanoparticles (Figure 4(A4)) were non-luminous, but they could turn green and blue sparkling completely after PF, PFTPE and PTTPE particles remained by ultrasonication treatment (Figure 4(B4–D4)) under the UV lamp (λ = 365 nm). Meanwhile, we could easily find that the foams skeleton decorated by PF, PFTPE and PTTPE, emitting bluish, bright grassy and yellowish green respectively under a fluorescence microscope in Figure 4(B3,C3,D3). As mentioned above, we can positively determine that the nanoparticles have been well loaded onto the sponge skeletons.

### 3.4. Comparison of Explosive Detection

**Figure 5:**
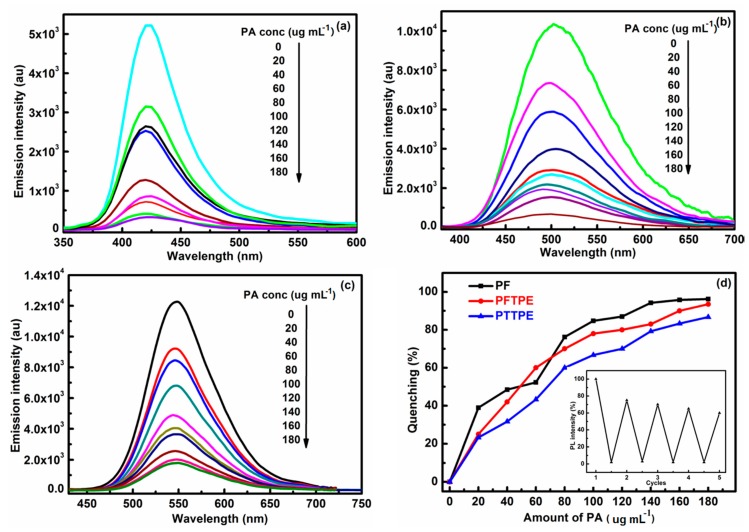
Fluorescence spectra of PU-PF (**a**), PU-PFTPE (**b**) and PU-PTTPE (**c**) in the presence of different PA concentrations (µg·mL^−^^1^); (**d**) Quenching efficiency-PA concentrations curve of PU-PF, PU-PFTPE and PU-PTTPE (µg·mL^−^^1^), inset: fluorescence recovery of used PU-PF after washed with ethanol.

The successful cases in the detection of explosives using fluorescent polymers or small molecules encourage us to explore the utility, as chemosensors, of these luminescent materials with different luminescence characteristics. With this in mind, we compared the detection effects of the ACQ, AIE and AEE polymers in the presence of PA. Picric acid (PA) was selected as an explosive pollutant not only due to its commercial availability but also because it was a strong explosive, as well as the pollution of PA in groundwater or soil caused severe health problems [43,44,45]. In general, the explosive molecules could be spread into the 3D reticular structure of PU foams as well as seized by the polymer nano-size particles when the sponges were put into PA solutions, and then the effective fluorescence quenching appeared. Figure 5 depicted the PL spectra of PU-PF (λ_em_ = 422 nm), PU-PFTPE (λ_em_ = 503 nm) and PU-PTTPE (λ_em_ = 554 nm) with the gradual addition of PA in methanol and the changes of the fluorescence intensity were recorded, meanwhile, the significant fluorescence quenching was observed in the PA solutions with different concentration. From the point of view, the comparative study to the practical application of the functionalized PU foams decorated by PF, PFTPE and PTTPE as chemosensors for explosive detection was performed.

As shown in Figure 5d, the chemical probes of these three polymers exhibited a very effective response to the nitro-aromatic explosive with different concentrations. PU-PF detected PA more effectively and sensitively than that of PU-PFTPE and PU-PTTPE at each of the same concentrations except 60 µg·mL^−1^, owing to the involvements of heterogeneous contact, self-absorption and/or energy transfer [46]. The fluorescence of PU-PF and PU-PFTPE could disappear completely at a PA concentration of 180 µg·mL^−1^, while PU-PTTPE still had a certain intensity of light yellow fluorescence. In addition, the quenching efficiencies of these three chemical probes were 96.2%, 93.5% and 86.7% at a PA concentration of 180 µg·mL^−1^, respectively. The photographs of functional foams at a PA concentration of 0 and 180 µg·mL^−1^, taken under the illumination of the UV lamp (365 nm) (Supplementary Materials Figure S8). With regard to Stern–Volmer formula (*I*_0_/*I*) = 1 + *K*_SV_ [Q] [47], the quenching constant (*K*_SV_) of PU-PF, PU-PFTPE and PU-PTTPE were calculated to be 29,087, 11,450 and 8270 M^−1^, respectively. Moreover, PU-PF still retained greater than 60% of the fluorescence intensity after four times recycles (Figure 5d), and the other two functional foams also showed this nature, meaning that PU-PF, PU-PFTPE and PU-PTTPE could be utilized multiple times after being washed by ethanol and dried at 60 °C in a vacuum oven overnight. From the results above, we could distinctly see that PU-PF detected nitro-aromatic explosive more effectively than PU-PFTPE or PU-PTTPE, and the good practical application of these three chemosensors based on PU foams was proved.

### 3.5. The Detection Principle of Sensors System

To further shed light on the quenching mechanisms of PU-PF, PU-PFTPE and PU-PTTPE, the quenching mechanism of photo-induced electron transfer (PET) was firstly discussed. It is easy to transfer electrons from multi electron conjugated polymers because PA is a kind of electron deficient compound. As shown in Table 1, the LUMO levels of PF, PFTPE and PTTPE were much higher than that of PA, which facilitated excited electron transfer from these three polymers to PA. It meant that PU-PF, PU-PFTPE and PU-PTTPE could detect nitro-aromatic explosive PA. Furthermore, the LUMO levels of PFTPE or PTTPE were lower than that of PF and the quenching efficiency of PU-PFTPE or PU-PTTPE were likely to be better than that of PU-PF. However, as seen from the quenching efficiency-PA concentrations curve (Figure 5d), PU-PF gave a quenching efficiency of 96.2%, slightly higher than PU-PFTPE (93.5%) and PU-PTTPE (86.7%) at a PA concentration of 180 µg·mL^−1^, indicating that the PET was not a single mechanism governing the quenching behavior.

**Figure 6:**
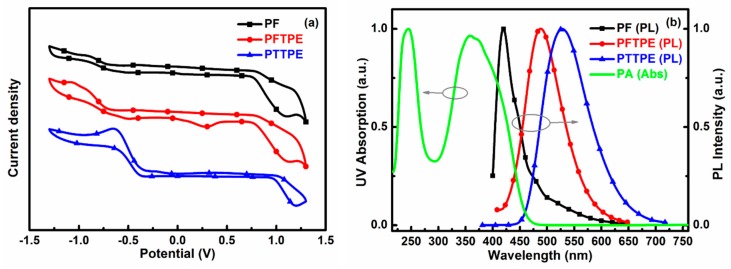
(**a**) Cyclic voltammetry curve and the energy levels of PF, PFTPE and PTTPE; (**b**) Absorption spectrum of PA, and the emission spectra of PF, PFTPE and PTTPE.

Secondly, another mechanism of fluorescence resonance energy transfer (FRET) was referred. As indicated in Figure 6b, there were overlaps between the UV absorption spectrum of an explosive (PA) and the emission spectra of the chemical sensors (PF, PFTPE and PTTPE). In contrast to that of PFTPE and PTTPE, the emission spectrum of PF was better overlapped with the absorption spectrum of PA, which was resulted from the effective energy transfer from the fluorophore (PF) to the nitro explosive (PA). This explanation was consistent with the previous experimental result. Consequently, two quenching mechanisms (PET and FRET) exist simultaneously, and the latter plays a more important role.

## 4. Conclusions

As mentioned previously, there have been few reports focus on a comparison of the ACQ, AIE and AEE polymers as probes to detect explosives under the same conditions. In general, we have successfully designed and synthesized three polymers with ACQ, AIE and AEE property, respectively, PF, PFTPE and PTTPE. Afterward, the functionalized PU foams decorated by these three polymers were prepared by ultrasonication method and utilized to detect explosives (PA). PU-PF, PU-PFTPE and PU-PTTPE exhibited higher quenching efficiencies, with 96.2% for PU-PF, 93.5% for PU-PFTPE and 86.7% for PU-PTTPE related to PA at 180 µg·mL^−1^. Interestingly, the quenching efficiency of ACQ-active PU-PF was higher than that of AIE-active PU-PFTPE or AEE-active PU-PTTPE by adopting this very common carrier (PU) and the preparation method (ultrasonication). At the same time, they can be recycled many times. Therefore, we believe that more optimized fabrication methods will improve sensing abilities of these fluorescent materials and it will further promote the development of fluorescent materials with high efficiencies.

## Figures and Tables

**Table 1:** The HOMO and LUMO levels of PA, PF, PFTPE and PTTPE.

Substance	HOMO (eV) ^2^	LUMO (eV) ^3^	Band Gap (eV) ^4^
PA ^1^	−8.29	−3.87	4.42
PF	−5.49	−2.54	2.95
PFTPE	−5.52	−2.59	2.93
PTTPE	−5.71	−3.14	2.57

^1^ The data of PA is based on reported literature [45,48,49]; ^2^ Calculated from the following formula: *E*_HOMO_ = −(*E*_ox_ + 4.8 − *E*_FOC_); ^3^ Obtained by subtracting the optical band gap from the HOMO energy level, *E*_LUMO_ = *E*_HOMO_ + *E*g; ^4^ Optical band gap from the absorption onset wavelength. (Supplementary Materials Figure S9).

## Supplementary Materials

The following are available online at http://www.mdpi.com/1424-8220/18/5/1565/s1, Figure S1: ^1^H NMR spectrum of PF in CDCl_3_. Figure S2: ^13^C NMR spectrum of PF in CDCl_3_. Figure S3: ^1^H NMR spectrum of PFTPE in CDCl_3_. Figure S4: ^13^C NMR spectrum of PFTPE in CDCl_3_. Figure S5: ^1^H NMR spectrum of PTTPE in CDCl_3_. Figure S6: ^13^C NMR spectrum of PTTPE in CDCl_3_. Figure S7: Photographs of PTTPE (100 µg·mL^−1^) in H_2_O/THF mixtures with different water fractions, taken under the illumination of the UV lamp (365 nm). Figure S8: The photographs of functional foams at a PA concentration of 0 and 180 µg·mL^−1^, taken under the illumination of the UV lamp (365 nm). Figure S9: UV-vis absorption in thin solid film.
