# Supplementary material for: A Comparison of ACQ, AIE and AEE-Based Polymers Loaded on Polyurethane Foams as Sensors for Explosives Detection
Source: Sensors (Basel). 2018 May 15;18(5):1565. doi: 10.3390/s18051565 (PMC5982694; doi:10.3390/s18051565)
Supplement: Supplementary file 1 [file sensors-18-01565-s001.pdf]

sensors

Supplementary Materials: A Comparison of ACQ, AIE and AEE-based Polymers Loaded on Polyurethane Foams as Sensors for Explosives Detection

Zhiwei Chu [1], Zhuxin Fan [1], Xiang Zhang [1], Xiaofeng Tan [2], Dongxu Li [1], Guohua Chen [1] and Qinghua Zhao [1,*]

[1] College of Materials Science and Engineering, Huaqiao University, Xiamen 361021, China; chuzhiwei1991@126.com (Z.C.); 1300507012@hqu.edu.cn (Z.F.); 1611302036@hqu.edu.cn (X.Z.); lidongxu@hqu.edu.cn (D.L.); hdcgh@hqu.edu.cn (G.C.)

[2] Department of Polymer Chemistry and Technology, Kaunas University of Technology, K. Barsauskog. 59, 51423 Kaunas, Lithuania; xiaofeng.tan.chn@gmail.com

* Correspondence: qhzhao@hqu.edu.cn; Tel.: +86-0592-6162225

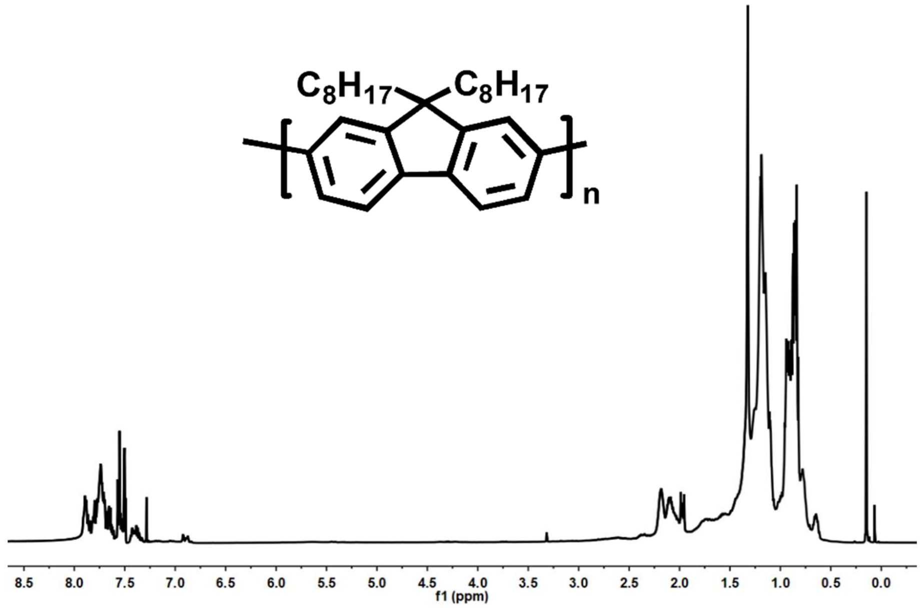

Figure S1. ^{1}H NMR spectrum of PF in $CDCl_3$.

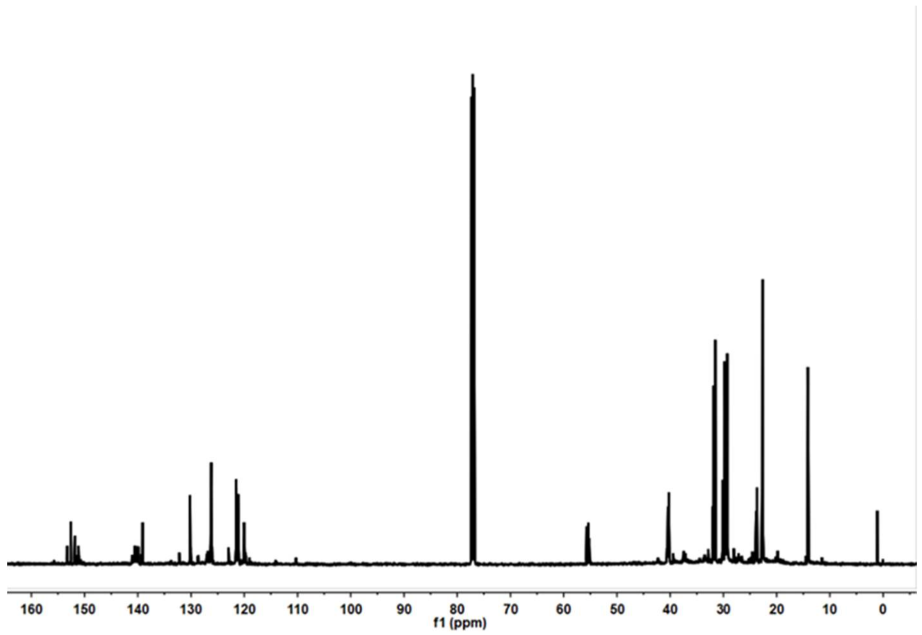

Figure S2. ^{13}C NMR spectrum of PF in $CDCl_3$.

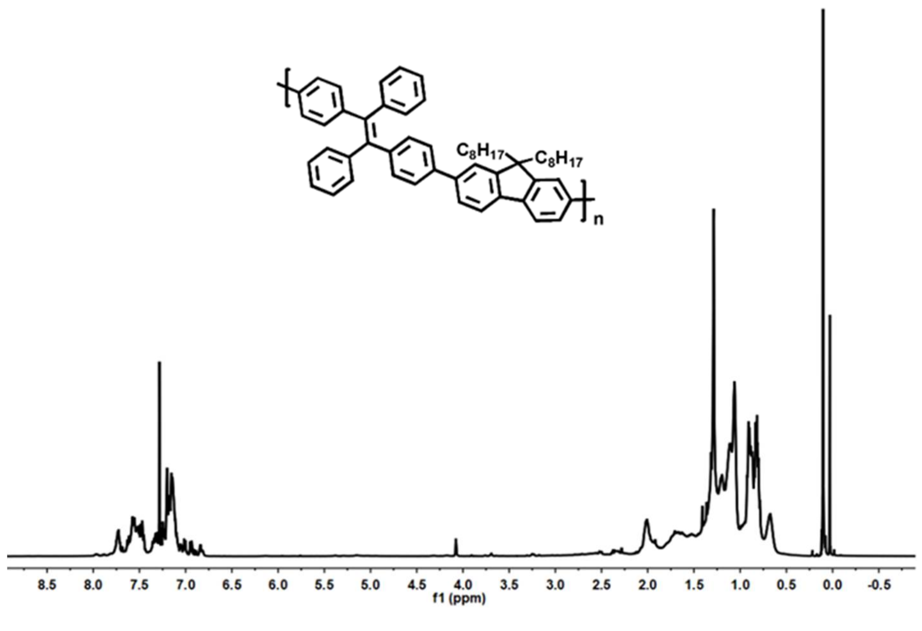

Figure S3. ^{1}H NMR spectrum of PFTPE in $CDCl_3$.

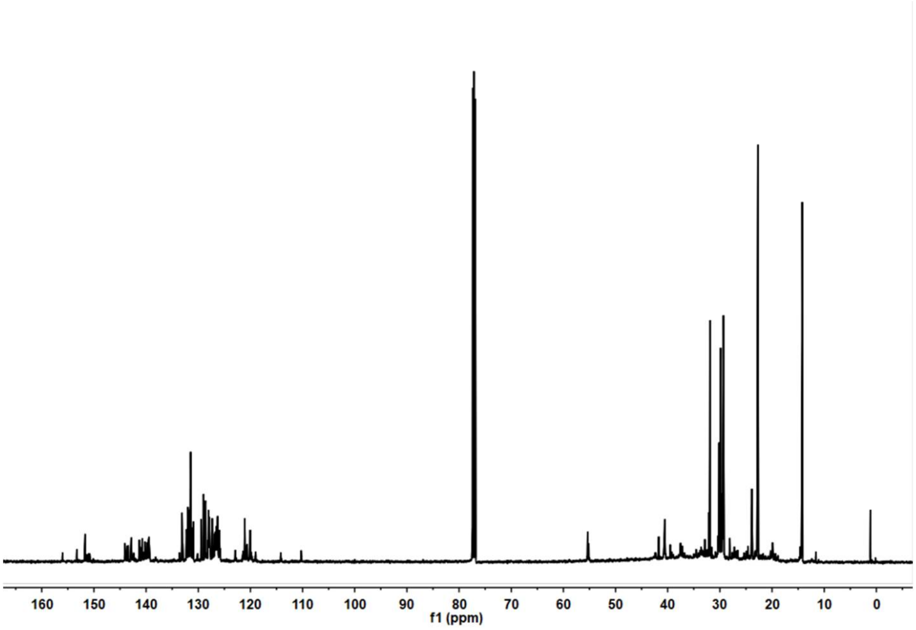

Figure S4. ^{13}C NMR spectrum of PFTPE in $CDCl_3$.

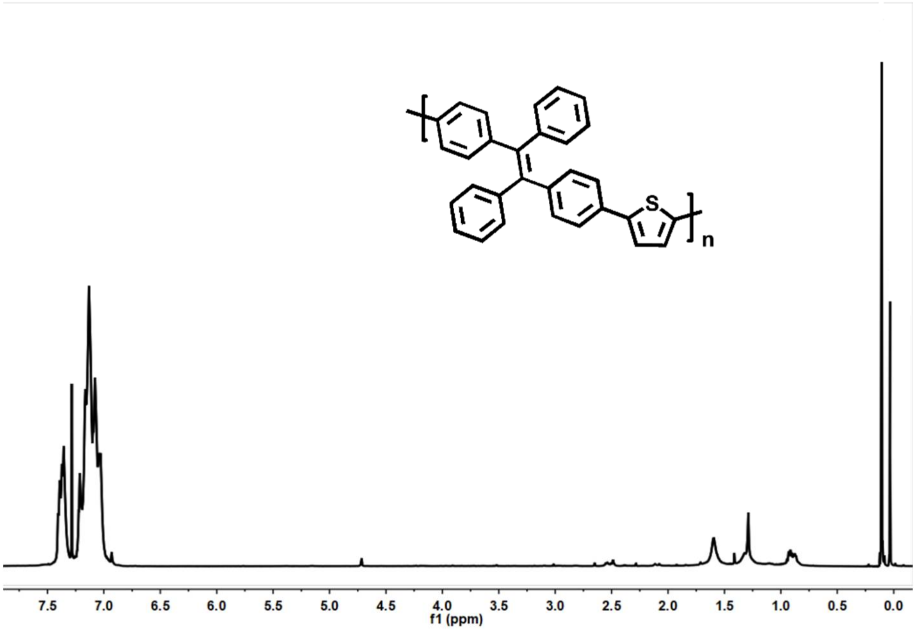

Figure S5. ^{1}H NMR spectrum of PTTPE in $CDCl_3$.

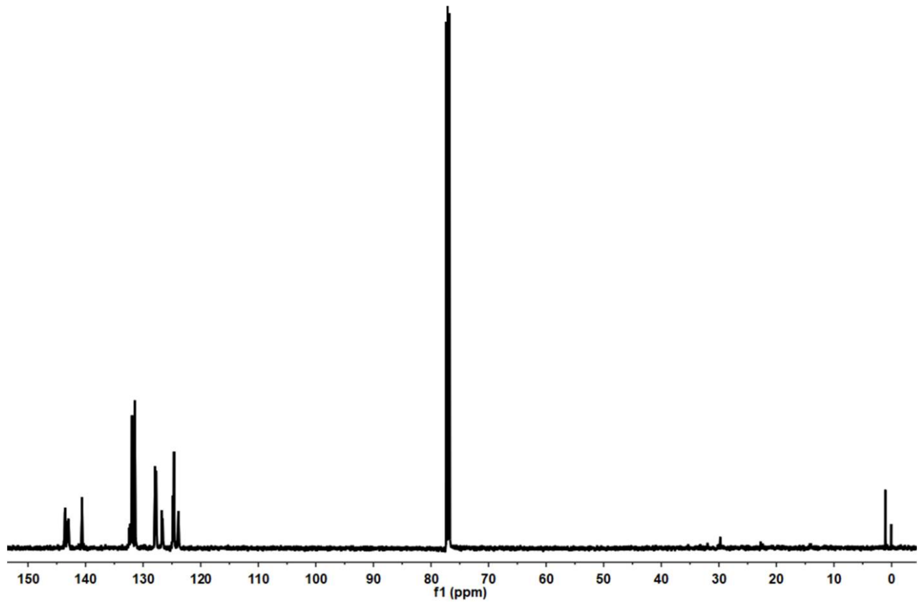

Figure S6. ^{13}C NMR spectrum of PTTPE in $CDCl_3$.

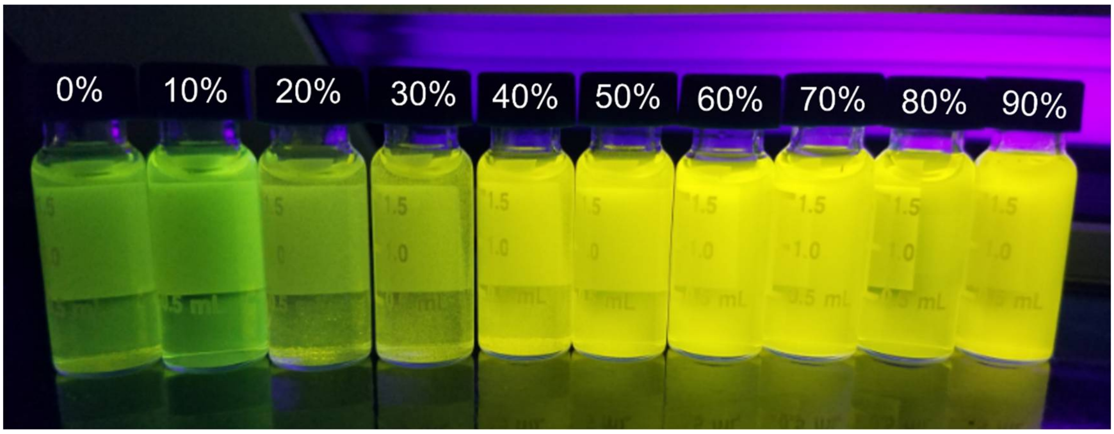

Figure S7. Photographs of PTTPE (100 μg·mL^{-1}) in H_2O/THF mixtures with different water fractions, taken under the illumination of the UV lamp (365 nm).

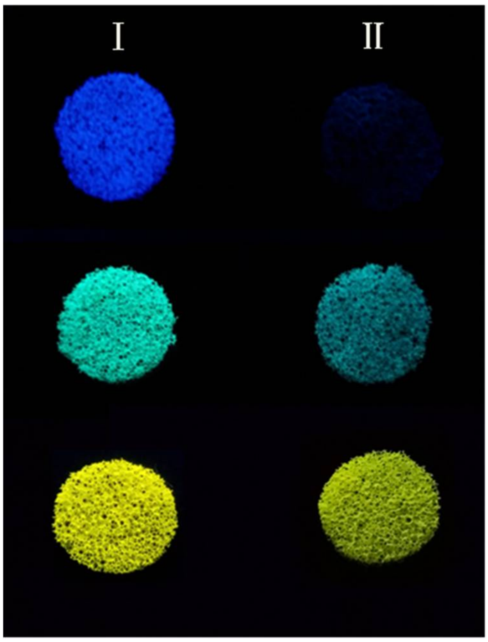

Figure S8. The photographs of functional foams at a PA concentration of 0 and 180 μg·mL^{-1}, taken under the illumination of the UV lamp (365 nm).

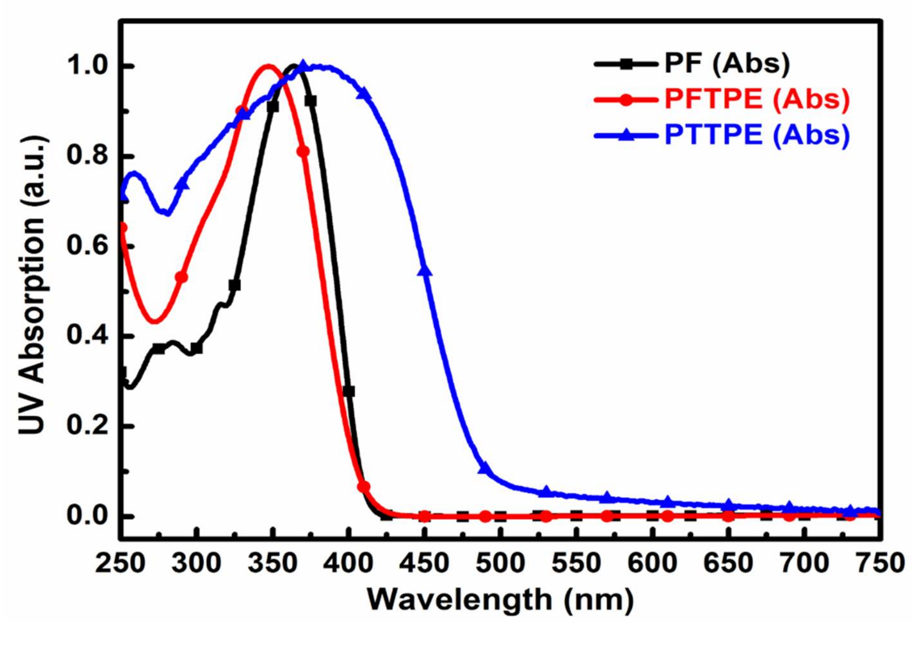

Figure S9. UV-vis absorption in thin solid film.
